# Haploidy and aneuploidy in switchgrass mediated by misexpression of *CENH3*

**DOI:** 10.1002/tpg2.20209

**Published:** 2022-04-25

**Authors:** Sangwoong Yoon, Jennifer Bragg, Sheyla Aucar‐Yamato, Lisa Chanbusarakum, Kurtis Dluge, Prisca Cheng, Eduardo Blumwald, Yong Gu, Christian M. Tobias

**Affiliations:** ^1^ USDA‐ARS, Western Regional Research Laboratory Albany CA USA; ^2^ Dep. of Plant Sciences Univ. of California Davis CA USA

## Abstract

Cross bred species such as switchgrass may benefit from advantageous breeding strategies requiring inbred lines. Doubled haploid production methods offer several ways that these lines can be produced that often involve uniparental genome elimination as the rate limiting step. We have used a centromere‐mediated genome elimination strategy in which modified *CENH3* is expressed to induce the process. Transgenic tetraploid switchgrass lines coexpressed Cas9, a poly‐cistronic tRNA‐gRNA tandem array containing eight guide RNAs that target two *CENH3* genes, and different chimeric versions of *CENH3* with alterations to the N‐terminal tail region. Genotyping of *CENH3* genes in transgenics identified edits including frameshift mutations and deletions in one or both copies of the two *CENH3* genes. Flow cytometry of T_1_ seedlings identified two T_0_ lines that produced five haploid individuals representing an induction rate of 0.5% and 1.4%. Eight different T_0_ lines produced aneuploids at rates ranging from 2.1 to 14.6%. A sample of aneuploid lines were sequenced at low coverage and aligned to the reference genome, revealing missing chromosomes and chromosome arms.

## Core Ideas

- Haploid switchgrass can be produced by a line with defective *CENH3*.
- Switchgrass *CENH3* genes are necessary for faithful chromosome segregation.
- Genome editing switchgrass *CENH3* results in mutations and deletions that can affect steady state RNA levels.

**Abbreviations**
DH: doubled haploid
PCR: polymerase chain reaction
PTG: poly‐cistronic tRNA‐gRNA
qRT‐PCR: real‐time quantitative reverse transcription polymerase chain reaction
T‐DNA: transfer DNA
WT: wild‐type

## 1 INTRODUCTION

Haploid technology has been effectively used in a wide variety of plants, advancing many aspects of basic biology and practical genetics (Forster et al., 2007). Haploid plants, whether derived from a diploid or a polyploid, have half the chromosome number of the euploid form. If the haploid genome is doubled (either spontaneously or via the application of anti‐mitotics like colchicine) the resulting doubled haploid (DH) lines are completely homozygous. When haploid production is efficient, the technique may be used in breeding programs to shorten the selection cycle and effort required to evaluate performance of new lines.

An in vivo centromere‐mediated genome elimination strategy has been used to produce haploid individuals in several plant species. First in Arabidopsis (Ravi & Chan, 2010), and recently in maize (*Zea mays* L.) and wheat (*Triticum aestivum* L.) (Lv et al., 2020; Wang et al., 2021). This approach and variations of it have demonstrated a critical role for *Centromeric Histone H3* (*CENH3*) in the fidelity of chromosome segregation during early embryogenesis. *CENH3* is a modified histone H3 that specifies the centromere and mediates interaction with the kinetochore apparatus. It is now evident that a wide variety of mutations in *CenH3* and *CenH3* null‐heterozygotes can act as haploid inducers (Karimi‐Ashtiyani et al., 2015; Kuppu et al., 2020; Wang et al., 2021). This occurs through missegregation and micronuclei formation leading to elimination of chromosomes whose centromeres are loaded with the variant CenH3 during early embryogenic cell divisions.

Another in vivo technique for haploid production that has been exploited in maize and recently transferred to rice and wheat relies on mutations in a pollen‐specific phospholipase *Matrilineal (MTL)*, also called *Not Like Dad* or *Phospholipase A1* (Gilles et al., 2017; Kelliher et al., 2017). This mutation is found in the maize haploid‐inducing inbred line called Stock 6 (Coe, 1959). Separate mutations in *ZmDMP*, a DUF679 domain membrane protein, can also cause genome‐elimination and enhances haploid induction frequency in an *mtl* background (Zhong et al., 2019).

In tetraploid switchgrass, we define haploid as genome‐copy loss from both subgenomes. Doubled haploid technology requires an efficient 2n‐inducing system along with subsequent genome doubling and would enable novel breeding opportunities such as the selection of high performing inbred lines for commercial hybrid production systems. Heterosis for biomass yield is seen in crosses between heterozygous parents from different switchgrass subpopulations (Bhandari et al., 2017; Martinez‐Reyna & Vogel, 2008; Vogel & Mitchell, 2008). However, inbred lines have not been developed due to genetic incompatibility systems, which are active in switchgrass as well as inbreeding depression and sterility that can occur in the relatively few selfed genotypes that are obtained. If better inbred lines were available, development of high yielding single‐cross hybrids would be an optional breeding approach. As the performance of inbreds is often correlated with the performance of their hybrids, selection of high‐yielding inbred lines may provide advantages (Hayes & Johnson, 1939; Sprague, 1977). In addition, DH technology would facilitate the introgression and stabilization of desired traits, alien genes, transgenes, chromosome segments, or whole chromosomes (Devaux & Pickering, 2005; Forster & Thomas, 2005).

The base chromosome number in switchgrass is nine with two subgenomes, N and K (Gould, 1975; Lovell et al., 2021), but the species is a complex of different cytotypes, with tetraploids (2*n* = 4*x* = 36) and octaploids (2*n* = 8*x* = 72) predominating (Brunken & Estes, 1975; Hopkins et al., 1996). Haploid (2*n* = 18) production in switchgrass via anther culture has been reported but was inefficient (Conger, 2002). We previously identified two haploid lines from among the progeny of a controlled cross between northern and southern lowland individuals (Young et al., 2010). These haploids can be easily distinguished from tetraploid individuals by their reduced stature, smaller epidermal cell size, and lower number of chloroplasts per guard cell. The haploids are sterile but can be maintained vegetatively. Their slow growth and poor performance have so far prevented large‐scale genome doubling experiments. However, colchicine‐induced genome doubling of tetraploid embryogenic calli, and seedlings has produced octaploids (Yang et al., 2014; Yoon et al., 2017).

Previously, two switchgrass genes (*CENH3‐1* and *CENH3‐2*) were identified from genomic scaffold sequences based on similarity to Arabidopsis *Histone H3*. One of these was demonstrated to be nuclear localized by fusion to yellow fluorescent protein and transient overexpression in *Nicotiana benthamiana* Domin. Leaf RNAseq data shows that both genes are transcribed (Miao et al., 2016) and the two genes are homeologs due to their sequence identity, local gene environments, and syntenic positions in the switchgrass reference genome with *CENH3‐1* present on chromosome 3N and *CENH3‐2* present on chromosome 3K (Lovell et al., 2021). In this work we set out to test whether CRISPR‐induced gene edits to these essential switchgrass genes could lead to generation of haploid inducer lines. Because of uncertainty about the specific role of each homeolog, we developed poly‐cistronic tRNA‐gRNA (PTG) array‐expressing constructs that were designed to target four functional domains in each homeolog that were co‐expressed along with Cas9 and different synthetic CENH3* gene fusions that have been demonstrated to induce haploidy in other species. Frequency of aneuploid and haploid generation was evaluated in the transgenic lines.

**Core Ideas**

- Haploid switchgrass can be produced by a line with defective *CENH3*.
- Switchgrass *CENH3* genes are necessary for faithful chromosome segregation.
- Genome editing switchgrass *CENH3* results in mutations and deletions that can affect steady state RNA levels.

## 2 MATERIALS AND METHODS

### 2.1 Plant material

A clonally propagated single genotype of the cultivar *Alamo*, designated ALBA4, that previously has been identified as highly transformable was used for all experiments (Saathoff et al., 2011). Because switchgrass is self‐incompatible, flowering transgenic lines were either pollinated with other switchgrass lines flowering at the same time under long‐day conditions in the greenhouse (open pollination) or were crossed with the genotype AP13 by securing micromesh fabric pollination bags (Vogel et al., 2014) over both genotype's flowering panicles.

All plants were grown in a greenhouse at the ARS Western Regional Research Center, in 13.64‐liter pots containing Sunshine Mix #1 growth media (Sun Gro Horticulture, Vancouver, CN). Plants were watered daily with a nutrient solution containing 100 ppm N via drip irrigation supplemented with Peters 20‐20‐20. The greenhouse was maintained between 18 °C and 27 °C under 24‐h light with supplemental LED lighting (Lumigrow, Emeryville, CA).

### 2.2 DNA cloning and plant transformation

Guide RNA design was carried out with the assistance of the CRISPOR tool (Haeussler et al., 2016) using regions from the predicted coding sequences and splice junction regions of *CenH3‐1* and *CenH3‐2*. The PTG self‐processing transcripts were prepared by synthesis using the methods described in detail elsewhere (Xie et al., 2015) with the plasmid pGTR and the primer sequences listed in Table S2 that were brought together via Golden Gate assembly (Engler et al., 2008). The PTG fusion was constructed in the order AE1–AE8 (Table 1) relative to the direction of transcription from the U3 promoter. The PTGs were then inserted into the unique BsaI site of plasmid pRGEB32. The resultant plasmid was then modified to create unique sites for insertion of a complementation gene cassette and to substitute strong monocot promoters for driving the selectable marker gene and for expression of Cas9. This was accomplished by first inserting an AscI/AvrII cloning adapter (Table S2) into the unique PmeI site of pRGEB32 such that the AscI site was closest to the right border transfer DNA (T‐DNA) sequence. Then, the maize ubiquitin promoter (Christensen & Quail, 1996) was amplified with primers *M Ubi pro FWD* and *M Ubi pro RVS* containing cloning sites Sbf1 and BstB1 and directionally cloned into the corresponding sites in the vector to drive expression of Cas9. Then, the rice *RUBQ2* polyubiquitin promoter (Wang et al., 2000) was substituted for the CaMV 35S promoter driving *HptII* coding sequences using unique HindIII and PspXI sites in both vector and insert DNAs. The resulting plasmid was sequence verified.

**TABLE 1:** *PviCENH3* CRISPR target sequences

Gene	Guide sequence	Guide sequence/protospacer adjacent motif	Target
*CENH3‐1*	AE01	CGCGGCCGTGAGGAAATCCA/*AGG*	Exon 1
	AE02	AGTTCGGGCGCTCCCCGAAC/*CGG*	Exon 1
	AE03	CGGGGAGCGGGCTTACCTGT/*CGG*	Exon 1‐Intron 1
	AE04	AAACCGCACCGTTGGAGGCC/*AGG*	Exon 4
*CENH3‐2*	AE05	CCTTCGATTTCCTCACGGCC/*GGG*	Exon 1
	AE06	CAGTTCGGGCGCTCCCCGCA/*CGG*	Exon 1
	AE07	CTCCGGTGAGTGCGTCCGAG/*CGG*	Intron 2
	AE08	AGGTGAAGAAACCACACCGT/*TGG*	Exon 4

The complementation gene expression cassettes were created by amplification of 2 kb of 5′ noncoding sequence from the *Pvi CenH3‐2* gene with *2N_Pvi_Pro_HMS Forward* and *2N_Pvi_Pro_HMS Reverse* primers, and then inserting it into the HindIII and SpeI sites of the pUbi‐BASK plasmid (courtesy of James Thomson), a modified pAHC20 plasmid (Christensen & Quail, 1996). The coding sequences of the complementation genes found in Pv_Imm31, Pv_Imm32, Pv_G75E31, Pv_GFP31, Pv_Zm31, Pv_Ath31 and Pv_GFP_TS were synthesized (Integrated DNA Technology) and inserted into the MfeI and SpeI sites of the vector. The whole expression cassette (CENH3‐2 promoter::complementation gene::Nos terminator) was then amplified with *Pvi cassette amp forward* and *Pvi cassette amp reverse* primers and inserted in the AscI/AvrII site of modified pRGEB32 plasmid at the AscI/AvrII adapter site.

Transformations were carried out via Agrobacterium co‐cultivation of inflorescence‐derived embryogenic callus using *A. tumefaciens* strain AGL1 (Lazo et al., 1991). Transformation procedures were identical to those described previously (Somleva et al., 2002). T_0_ lines were confirmed for the presence of T‐DNA with the primers: *hyg left*, *hyg right*, *CAS9 400 FWD*, and *CAS9 400 RVS* listed in Table S2.

### 2.3 Genotyping/real‐time quantitative reverse transcription polymerase chain reaction (qRT‐PCR)

Sequencing was performed to screen T_0_ lines for the presence of genome edits induced by single‐guide RNAs (sgRNAs) and Cas9. Regions spanning 94 and 98 bp upstream to 469 and 291 bp downstream of the CRISPR target sites of *CENH3‐*1 and *CENH3‐*2, respectively, were amplified with gene‐specific primers (Figure S2). Polymerase chain reaction (PCR) products were evaluated using Sanger sequencing, and traces were analyzed using the Synthego ICE CRISPR Analysis tool (www.synthego.com; Brinkman et al., 2014; Hsiau et al., 2019). Expression of transgenes was measured using qRT‐PCR. After extraction of total RNA from leaf tissue with the RNeasy Plant Mini Kit (Qiagen), qRT‐PCR was conducted on an Applied Biosystems Quant Studio 6 Flex instrument using Brilliant II SYBR Green QRT‐PCR Master Mix (Agilent Technologies). Samples were analyzed using QuantStudio software relative to a *GAPDH* gene (Gimeno et al., 2014). Sequences for primers used in genotyping and qRT‐PCR are provided (Table S2).

### 2.4 Flow cytometry

Procedures described previously (Arumuganathan & Earle, 1991) were used to determine DNA content per cell using nuclei stained with propidium iodide. The prepared material was analyzed using a BD Accuri C6 flow cytometer with fluorescence collected through a 610/30 bandpass filter (Omega Optical). Flow cytometric analysis of integral peak, log fluorescence, and forward scatter was performed. The mean DNA content per sample was based on at least 1,000 nuclei. The internal standard used for comparison was rice cultivar Nipponbare with a 2C genome content of 0.9 pg as estimated by flow cytometry and using the average value of 980 Mbp  =  1 pg (Bennett et al., 2000; Cavalier‐Smith, 1985; Uozu et al., 1997).

### 2.5 Root tip squashes

For chromosome counts, root tips approximately 0.5 cm long were collected and prepared using methods described by (Jenkins & Hasterok, 2007). Briefly roots tips were pretreated in 0 °C deionized water for 12 h and were then fixed either for 4 h or overnight in 3:1 ethanol/glacial acetic acid and stored at −20°C until use. Slide preparations were made by digesting the root tips for 30‐60 min at 37 °C in 50 g L^−1^ Onuzuka R‐10 cellulase and 30 g L^−1^ Macerozyme R‐10 (Phytotechnology Labs). Digestion time varied according to the thickness and degree of lignification of the roots. Squashes were dehydrated after coverslip removal and mounted in Vectashield (Vector Laboratories) containing 1.0 μg ml^−1^ 4′,6‐diamidino‐2‐phenylindole (DAPI) prior to visualization with an Olympus BX51 fluorescence microscope equipped with a 100x oil‐immersion objective.

### 2.6 Aneuploid sequencing analyses

Genomic DNA was extracted from young leaf tissue of the ALBA4 WT plant and selected CENH3 mutant aneuploid lines using a previously described CTAB method (Xin & Chen, 2012). Library preparation from randomly sheared genomic DNA selected for a 350‐bp insert size and Illumina Novaseq PE150 sequencing was performed at CD Genomics. Sequence quality was checked using FastQC (Andrews, S, 2010), and sequences were filtered using Trimmomatic v 0.39 (Bolger et al., 2014) to remove low quality reads and adapter sequences. Filtered sequences were aligned to the hardmasked Alamo AP13 *Panicum virgatum* v5.0 reference genome using bowtie2 v2.4.2 with default settings (Langmead & Salzberg, 2012). The genome was binned into 100‐kb segments, and the number of reads aligning to each segment were counted using BEDtools v 2.29.1 (Quinlan & Hall, 2010). Counts were represented as a percent of ALBA4 counts aligning to the corresponding regions, and the differences in the numbers of aligned reads between ALBA4 and each aneuploid line were plotted across the genome to identify underrepresented genome regions.

### 2.7 Statistical analyses

Linear modeling of seed weight, germination rate, and C‐value measurement data was performed using R version 4.0.5 (R Core Team, 2018), lme4_1.1‐27.1 (Bates et al., 2015), and emmeans_1.6.1 (Lenth, 2021). *P* values for pairwise comparisons were calculated using Tukey's honestly significant difference test.

## 3 RESULTS

### 3.1 Generation of switchgrass transformants with gene editing constructs

We chose the switchgrass genotype ALBA4 derived from the cultivar *Alamo* for this work due to its high transformability. To assist with design of sgRNA and later genotyping the results of editing activity the CENH3 genes were sequenced in this background. Short reads available from the ALBA4 genotype were aligned to Pavir.3NG079613/*CENH3‐1* and Pavir.3KG376200/*CENH3‐2* regions of the *Panicum virgatum* v5.1 reference genome and sequence variants were annotated. Within the predicted coding sequences of ALBA4 *CENH3‐1*, three of the four predicted variant sites were heterozygous for nonsynonymous mutations, while the fourth was heterozygous for a silent mutation. In the ALBA4 *CENH3‐2* coding sequence, there were nine heterozygous nonsynonymous and six heterozygous synonymous substitutions. A summary of the changes is presented in Table S1. The ALBA4‐specific consensus sequences and phase‐informative short reads provided sufficient information to partially phase the genes and design specific sgRNAs.

**FIGURE 1:**
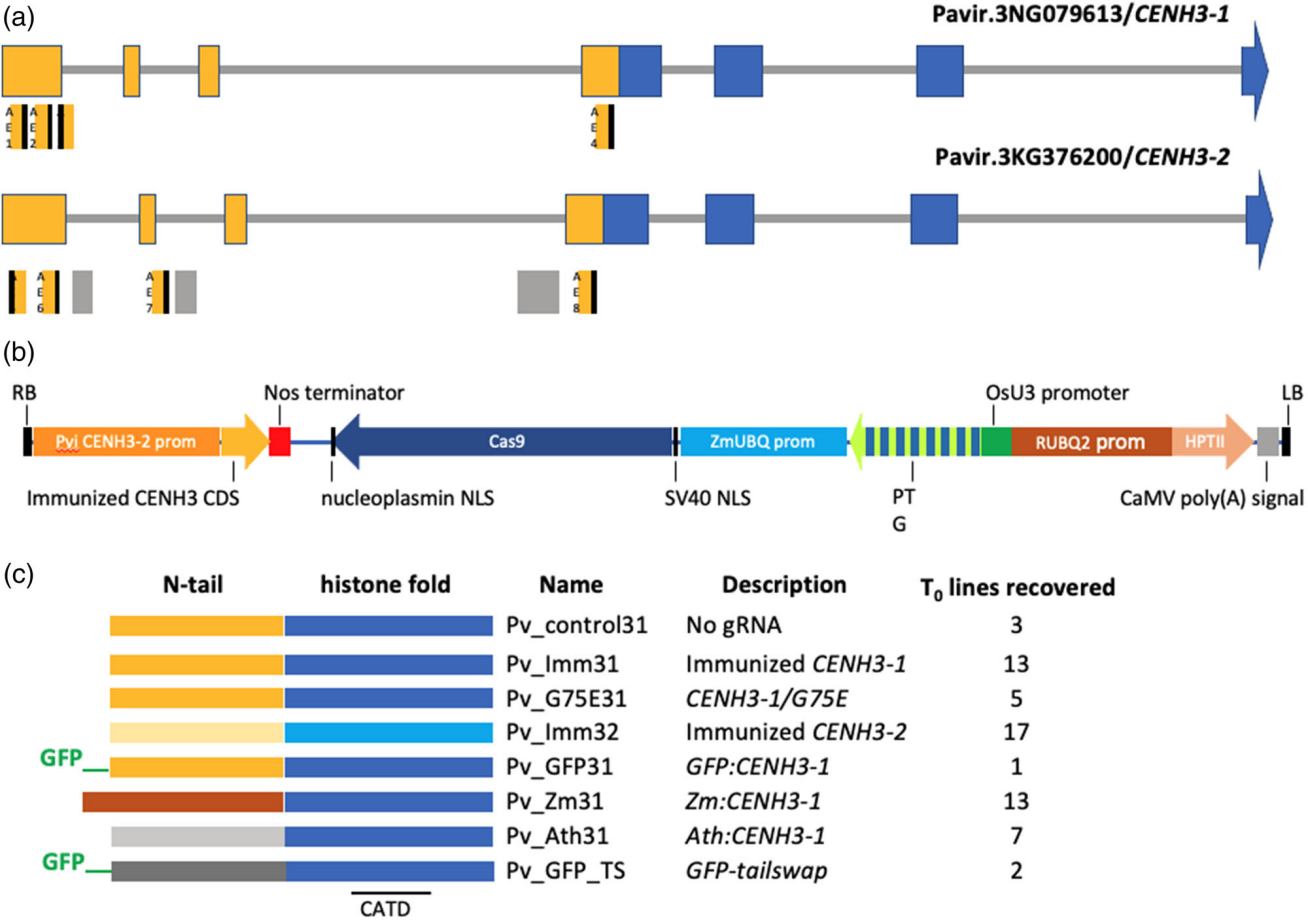
Pvi *CENH3* genes and transformation constructs. (a) Switchgrass *CENH3* gene structures with yellow and blue boxes representing exons and gray line spanning introns. Yellow and blue colors of exons represent the N‐terminal tail and histone fold domain respectively. Locations of sgRNA target sequences are marked by yellow blocks and protospacer adjacent motif (PAM) sequences by black lines below genes. Microsatellite regions of *CENH3‐2* are indicated by gray blocks. (b) Transformation construct transfer DNA (T‐DNA) region. Elements are labeled. RB and LB designate right and left T‐DNA borders, respectively. (c) Structure of complementation genes included in transformation constructs. Gene colors indicate sequence species of origin and divisions between N‐tail and histone fold regions. Switchgrass sequences are represented in orange and blue tones. Maize sequence is brown and *Arabidopsis* sequence is light gray. *P. virgatum* conventional histone H3 is indicated in dark gray. An asterisk indicates the position of the G75E mutation. N‐terminal GFP fusions are shown in green font. CATD centromere targeting domain is marked by the black line at bottom

A CRISPR Cas9 mutagenesis approach was used to target four sites in the predicted coding sequences and splice junction regions for both of the ALBA4 *CENH3* homoeologs (Figure 1a and Table 1). Sequence divergence between the two homoeologs prevented the design of sgRNA molecules that could target both simultaneously. In total, eight sgRNA sequences were expressed as a PTG controlled by the OsU3 promoter from the rice U3 small nuclear RNA to facilitate cleavage and assembly of the individual sgRNAs after transcription (Figure 1b). As controls and to mitigate the potentially lethal effects of double knockout mutations in one or both homoeologs, seven different CENH3* synthetic variants of *CENH3‐1* or *CENH3*‐*2* were created. Different binary plant‐transformation vectors individually co‐expressed CENH3* synthetic variants on the same T‐DNA with the PTG array under the control of *CENH3‐2* promoter sequence from the AP13 genotype. These complementation genes were immunized in the sense that they were synthesized to avoid complementarity to the sgRNA sequences present on the same T‐DNA (Figure 1c and Figure S1). The transformation constructs therefore each contained the same PTG array coupled to CENH3* coding sequences for: CENH3‐1 (Pv_control31/Pv_Imm31); CENH3‐2 (Pv_Imm32); a CENH3‐1/G75E modified protein (Pv_G75E31); an N‐terminal fusion of GFP to CENH3‐1 (Pv_GFP31); a chimeric fusion encoding GFP fused to the N‐terminal tail of conventional *P. virgatum* histone H3 followed by the histone fold domain of CENH3‐1 analogous to the original *GFP‐tailswap* used by Ravi and Chan (2010) (Pv_GFP_TS); a chimeric fusion encoding the N‐terminal hypervariable tail region from maize CENH3 joined to the histone fold region of CENH3‐1(Pv_Zm31); and finally, the N‐terminal hypervariable tail region from ArabidopsisCENH3 joined with the histone fold region of CENH3‐1 (Pv_Ath31). The Pv_control31 construct served as a control containing the immunized *CENH3‐1* gene but lacking the PTG array.

Variable numbers of regenerants were recovered for the individual transformations (Figure 1c) that were confirmed by PCR for the presence of the *HptII* resistance gene and *Cas9*. The constructs encoding WT CENH3‐1 or CENH3‐2 protein sequences, Pv_Imm31, Pv_Imm32, and the maize N‐tail swap protein, Pv_Zm31, yielded >10 lines each. Fewer lines were recovered from constructs encoding the switchgrass CENH3‐1 point mutant (Pv_G75E31), the Arabidopsis N‐tail swap protein (Pv_Ath31), and the GFP containing proteins (Pv_GFP31 and Pv_GFP_TS), despite multiple transformation attempts.

### 3.2 Transgenic lines carry multiple CENH3‐1/CENH3‐2 edits and are chimeric

To detect sequence alterations such as deletions and other potentially consequential mutations affecting one or multiple target sites, regions spanning ∼100 bp upstream and > 200 bp downstream of the CRISPR‐targeted sites were amplified from genomic DNA and directly sequenced. Primers used to amplify and sequence CENH3 target regions were designed to avoid microsatellites in the *CENH3‐2* gene (Figure 1a, Figure S2). Larger deletions were discovered and delineated through amplification of distant primers, however some unamplified large structural changes in a single copy of either *CENH3‐1 or 2* may appear identical to a homozygous mutation or wild‐type. Upon sequencing, amplicons from genotyped samples produced either clean sequence trace data that was easily characterized or overlapping sequence traces that required deconvolution with Synthego ICE software designed to infer CRISPR edits and quantitatively assess representation of WT and edited sequence in the samples. We interpreted results showing >25% edited sequences as heterozygous mutant and >75% as homozygous mutant with two distinct alleles. However, some ambiguity in trace deconvolution remained present due to small percentages of other sequences reported by the software arising from noisy trace data.

Collectively, the genotyping of T_0_ lines uncovered edits occurring in each of the eight targeted regions, validating the design of the CRISPR guide sequences and the activity of CAS9 in the switchgrass lines. Mutations that were detected consisted of point mutation, small indels, and larger deletions of >300 bp deletions (Table S3 and S4). As expected, the sequences of either CENH3 homeolog from the Pv_control31 control lines were unedited. Few edits were seen in Pv_Imm31, Pv_G75E31, and Pv_GFP31 lines, and these primarily represented heterozygous single‐base indels and point mutations that did not result in frameshifts. Much higher editing activity was detected in Pv_Imm32, Pv_Zm31, Pv_Ath31, and Pv_GFP_TS T_0_ plants and was more frequently observed for *CENH3‐2* sequences over *CENH3‐1* sequences (Tables S3 and S4). Among lines generated using these four constructs, 17 contained homozygous mutations predicted to negatively affect protein function of one homeolog. Of these, seven also presented heterozygous edits in the other homeolog and four contained mutations predicted to negatively affect protein function of both homeologs (Tables S3 and S4).

Genetic uniformity was assessed as well by sequencing PCR products from 3 to 10 individual tillers of each T_0_ line. Initial genotyping of multiple shoots from the same regenerants indicated several T_0_ lines were chimeric, as would be expected if target site cleavage and nonhomologous end joining DNA repair were recurrent or occurred in a subpopulation of cells during regeneration stages (Table S4). Therefore, we decided to classify every line as potentially chimeric, knowing that further crosses would be required to identify null T‐DNA segregants with desired *CENH3* mutations at a later point in time. However, as frameshifted edits and large deletions are unlikely to revert to WT, we analyzed a subset of clonally propagated, individual Pv_Zm31 and Pv_GFP_TS T_0_ with predicted deleterious mutations derived from single axillary buds. Genotypes of the cloned lines and predicted protein effects are shown in Tables 2 and 3.

**TABLE 2:** Edits fixed in cloned Pv_Zm31 and Pv_GFP_TS lines

Line	*CENH3‐1*	*CENH3‐2*
Pv_Zm31‐2A	No edits	AE08 HM 1 bp insertion
Pv_Zm31‐6A	AE01‐AE03 ∼ 72 bp HT deletion	AE08 HM 21 bp deletion
Pv_Zm31‐8A	AE04 353 bp HT deletion	AE08 HM 1 bp insertion
Pv_Zm31‐12A	AE01 ∼24 bp HT deletion	AE05‐AE06 ∼ 53 bp HM deletion; AE08 HM silent mutation
Pv_GFP_TS‐3A	AE01 1 bp HM insertion; AE03 6 bp HM deletion; AE04 1 bp HM deletion	AE06 HM 1 bp insertion; AE08 HM silent mutation

*Note*. HT, heterozygous; HM, homozygous.

**TABLE 3:** Protein effects predicted in cloned Pv_Zm31 and Pv_GFP_TS T_0_ lines

	CENH3‐1			CENH3‐2		
Line	Length (aa)	Edits	Predicted protein changes	Length (aa)	Edits	Predicted protein changes
A4	168	NA	WT^a^	168	NA	WT
Pv_Zm31‐2A	168	none	WT	106	HM	FS after codon 70, stop at codon 107
Pv_Zm31‐6A	147^b^	HT	FS after codon 11,^b^ disrupts GT intron splicing sequence	161	HM	deletes Arg71 to Val 77, maintains frame
Pv_Zm31‐8A	102	HT	FS after codon 61, stop at codon 103	106	HM	FS after codon 70, stop at codon 107
Pv_Zm31‐12A	57	HT	FS after codon 11, stop at codon 58	88	HM	FS after codon 8, stop at codon 89
Pv_GFP_TS‐3A	167	HM	FS codons 12 to 73 followed by 2 bp deletion that restores read frame	106	HM	FS after codon 26, stop at codon 107

*Note*. FS, frameshift; HT, heterozygous; HM, homozygous.

^a^ Identical to ALBA4 sequence, wild type (WT).

^b^ Reflects length if intron is spliced as in the WT gene.

### 3.3 Endogenous CENH3‐1/CENH3‐2 RNAs are reduced and CENH3* RNAs are detectable in transgenic lines

**FIGURE 2:**
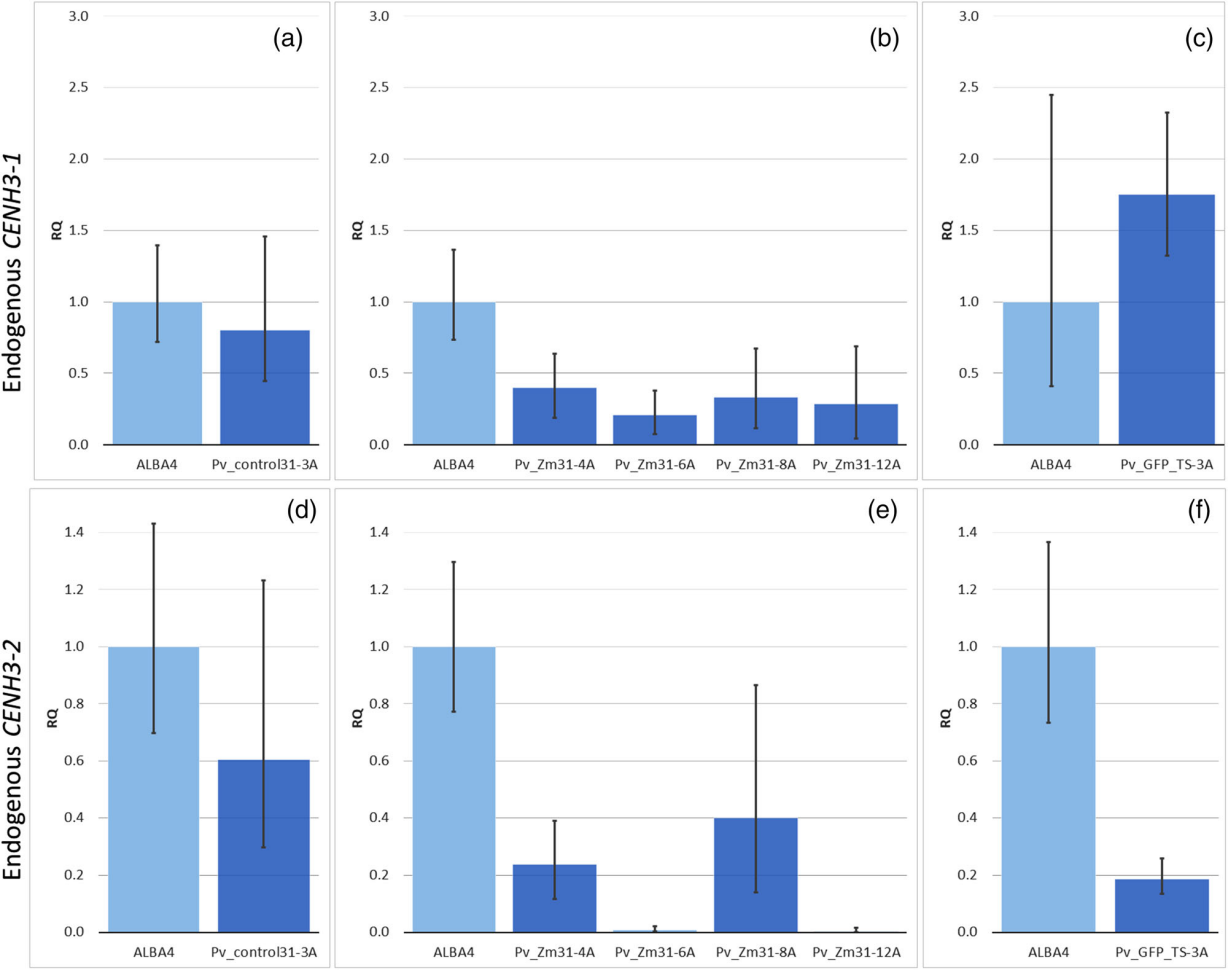
Expression of endogenous *CENH3* genes in leaf tissue of transgenic lines. Plots show gene expression in transgenic lines as relative quantities (RQ) of endogenous CENH3‐1 (a–c) and CENH3‐2 (d–f) compared with a ALBA4 WT plant. Error bars show the full range of expression measured

Expression of endogenous *CENH3* homeologs and CENH3* transgenes were measured in cloned Pv_Zm31 and Pv_GFP_TS T_0_ plants and the Pv_control31 control line using qRT‐PCR (Figure 2 and Figure S3) to ascertain if editing results in RNA misregulation. CENH3* transgene leaf expression was detected in all tested lines (Figure S3). The Pv_control31‐3A control line displayed similar expression to ALBA4 for both *CENH3* homeologs (Figure 2a and d), while the Pv_Zm31 lines with *CENH3‐1* heterozygous frameshift edits each show *CENH3‐1* expression approximately half of that observed in the wild‐type ALBA4 (Figure 2b). Pv_GFP_TS‐3A harbors homozygous null mutations in *CENH3‐1*. However, these edits alter codons 12 to 73 without causing a premature stop or substantially affecting qRT‐PCR primer binding sites, and transcript levels are on par with the nontransgenic control (Figure 2c). All four lines contain homozygous mutations in *CENH3‐2*, and expression is essentially undetectable in the Pv_Zm31‐6A and Pv_Zm31‐12A lines with > 20 bp deletions. Lines Pv_Zm31‐4A, Pv_Zm31‐8A, and Pv_GFP_TS‐3A contain smaller 1–2 bp frameshift edits that result in stop codons upstream of the reverse primer annealing site, and *CENH3‐2* expression is reduced but still detectable (Figure 2e‐f).

### 3.4 Partial and complete genome elimination among progeny of select T_0_ transgenic lines

**FIGURE 3:**
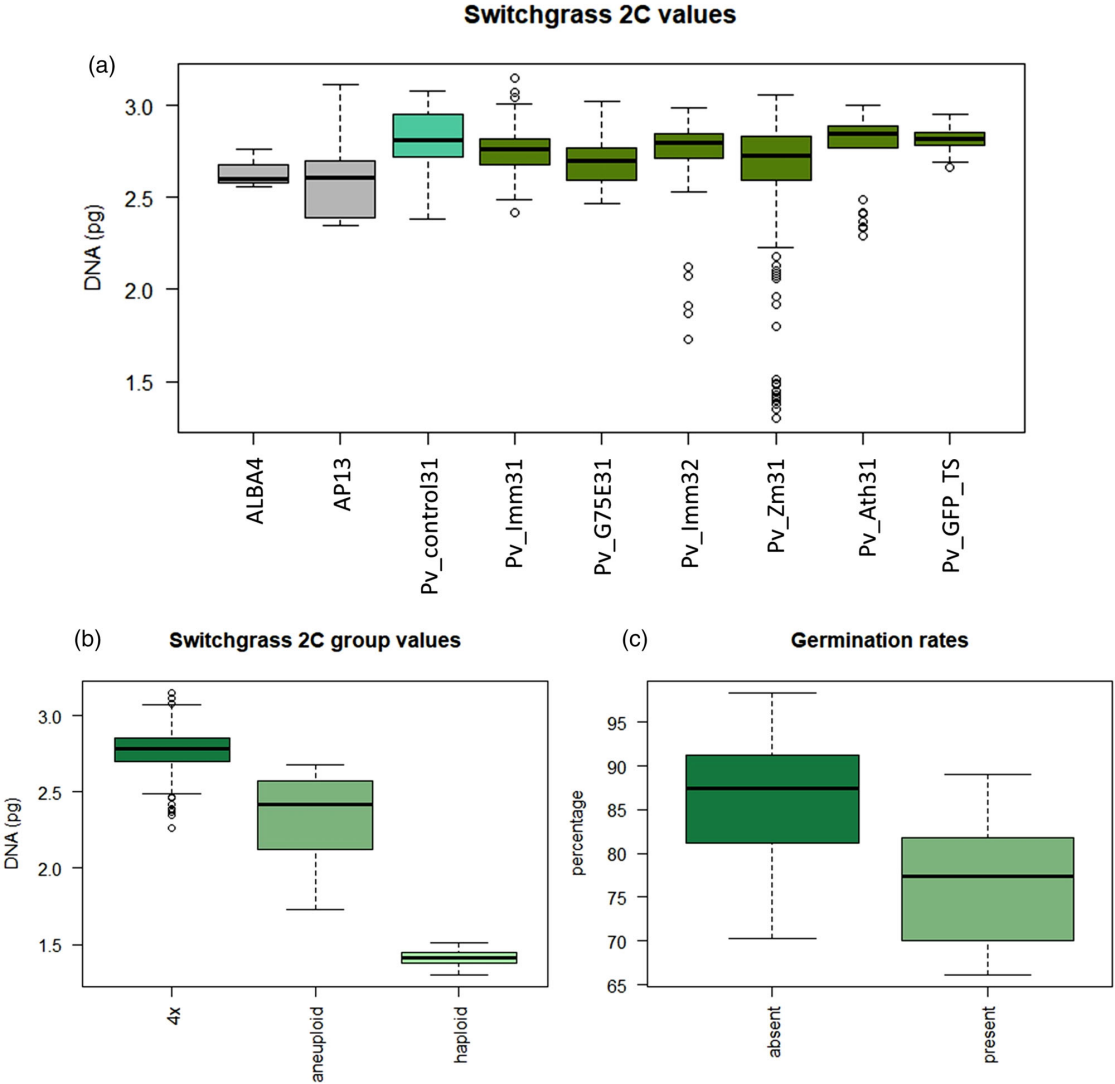
C‐value measurements of wild‐type lines and CENH3* T_1_ seedlings. Whisker plots illustrate the average 2C‐values for (a) AP13 and ALBA4 WT plants (gray) and all T_1_ progeny of the transgenic Pv_control31 control (turquoise) and CENH3 T_0_ mutant lines (green). Groups of Pv_Imm32, Pv_Zm31, and Pv_Ath31 low measurements represent putative aneuploid and haploid individuals. (b) Tetraploid (4x) T_1_ compared with aneuploid and haploid seedlings. (c) Germination rates for seeds from CENH3* lines from which aneuploid progeny were identified and lines from which no aneuploids were found

To survey partial and whole genome elimination rates, C‐values were estimated from seedlings derived by either open pollination in the greenhouse or controlled crosses. As switchgrass normally exhibits a high degree of self‐incompatibility, the assumption of minimal self‐pollination was made. Open greenhouse pollination of Pv_control31, Pv_Imm31, Pv_G75E31, Pv_Imm32, Pv_Zm31, Pv_Ath31, and Pv_GFP_TS transgenics and controlled crosses of Pv_Zm31‐6A collectively produced > 4000 seeds of which 82.1% germinated and grew sufficiently to estimate C‐values. Initial samples comprised pools of 2–10 plants, and individuals within a pool were subsequently re‐screened when flow histograms produced a population of nuclei with lower C‐values. An average 4x = 2C value of 2.77 pg was estimated from > 400 pooled reference line and T_1_ seedling samples. Family means for T_1_ seedlings ranged between 2.74‐2.87 pg (Figure 3a) and were ∼6% higher than but not significantly different from the AP13 (2.63 pg) and ALBA4 (2.64 pg) means (Table S5).

Pv_control31, Pv_Imm31, and Pv_GFP_TS lines showed no evidence of inducing genome reduction/elimination (Table 4) despite a likely double knockout of *CENH3‐1/CENH3‐2* in Pv_GFP_TS‐3a (Figure 3a and Table 3). The other lines showed varying abilities to generate aneuploids (Table 4). Notably, four of the Pv_Zm31 lines generated 72% of the identified aneuploids. Furthermore, Pv_Zm31‐6A and Pv_Zm31‐8A produced the only seedlings whose 2C‐values indicated full genome elimination (haploids). These cases were relatively infrequent, representing 0.5 and 1.4% of screened seedlings respectively. The values in Table 4 include seedlings produced from two controlled crosses of Pv_Zm31‐6A with AP13 that we performed in order to assess both male and female sides of the cross. (Table S6). A total of 1,000 seedlings were screened (250 from each side of two crosses), and the 12 aneuploids and four haploids detected were where Pv_Zm31‐6A served as the maternal parent. All progeny from the AP13 female parent had 2C‐values similar to 4x controls, and seedlings from this side of the cross were not included in the totals listed in Table 4.

**TABLE 4:** Summary of aneuploid production

Line	Estimate of individuals tested	No. of aneuploids	% aneuploids	No. of haploids	% haploids
Pv_control31‐3A	144	0		0	
Pv_Imm31‐5A	48	0		0	
Pv_Imm31‐8A	48	0		0	
Pv_Imm31‐10A	48	0		0	
Pv_Imm31‐12A	48	0		0	
Pv_Imm31‐14A	48	0		0	
Pv_Imm31‐22	48	0		0	
Pv_G75E31‐1A	144	8	5.6%	0	
Pv_Imm32‐3B	48	5	10.4%	0	
Pv_Imm32‐4A	48	0		0	
Pv_Imm32‐6A	48	0		0	
Pv_Imm32‐7A	48	0		0	
Pv_Imm32‐9B	48	0		0	
Pv_Imm32‐11A	48	1	2.1%	0	
Pv_Imm32‐16A	48	0		0	
Pv_Imm32‐18A	48	0		0	
Pv_Zm31‐3A	48	0		0	
Pv_Zm31‐4A	88	8	9.1%	0	
Pv_Zm31‐6A	770	40	5.2%	4	0.5%
Pv_Zm31‐8A	72	4	5.6%	1	1.4%
Pv_Zm31‐12A	80	2	2.5%	0	
Pv_Ath31‐2A	48	0		0	
Pv_Ath31‐9A	48	7	14.6%	0	
Pv_GFP_TS‐1A	56	0		0	
Pv_GFP_TS‐3A	96	0		0	

Compared with the tetraploids, aneuploid and haploid groups had significantly lower 2C‐values (*p* < .001) of 2.26 pg and 1.36 pg, respectively (Figure 3a and 3b, Table S5). In addition, significantly lower germination (*p* < .01) occurred in seed lots that contained aneuploid seedlings (77.1%) compared with lots from which no aneuploids were identified (86.6%) (Figure 3c, Table S5). Seed size, weight, and total seed/panicle were not affected by the direction of the cross (data not shown).

**FIGURE 4:**
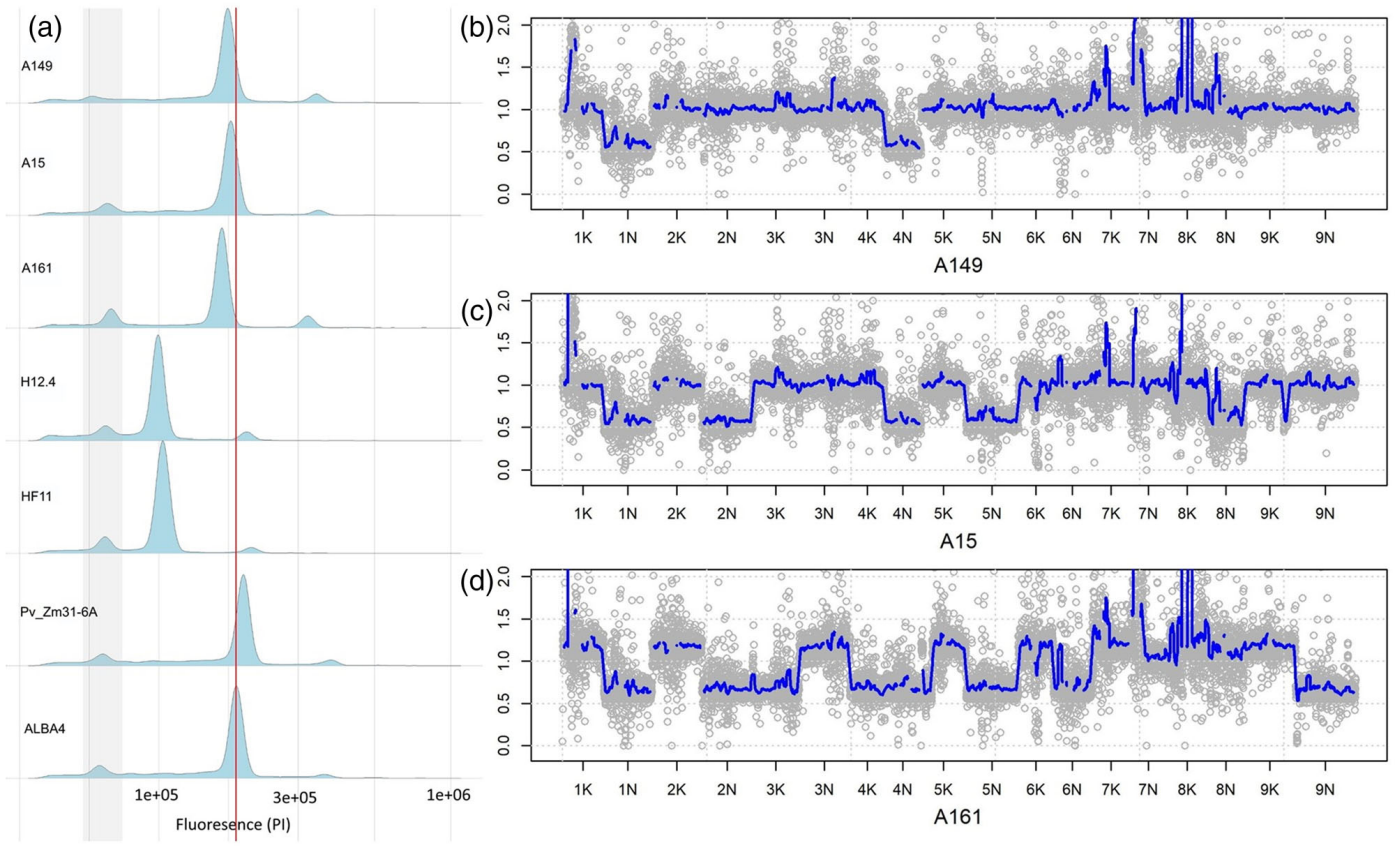
Aneuploidy among T_1_ seedlings. (a) Flow histogram with nuclei counts (y axis) and fluorescence (x axis) of switchgrass samples from top to bottom: T_1_ aneuploid lines A149, A15, A161, and T_1_ haploid lines H12.4 and HF11 derived from the cross Pv_Zm31‐6A x AP13, parental line Pv_Zm31‐6A, control line ALBA4. The red line indicates the location of the ALBA4 peak for comparison with other lines. Smaller left‐most histogram peaks represent nuclei in the G2 phase of the cell cycle. Histogram peak of the internal rice standard used for C‐value calculations is indicated by the gray shaded region on the right. (b–d) Read counts aligned along the switchgrass reference genome scaffold chromosomes and plotted in bins of 100 kbp. Individual bins indicated by gray points. Simple moving average of 50 bins represented by blue points. Data is represented as a fraction of ALBA4 counts. (b) Aneuploid A149. (c) Aneuploid A15. (d) Aneuploid A161

Low‐coverage sequencing obtained from several aneuploids agreed with flow cytometry data while providing detailed information on missing chromosomes. Figure 4a shows flow histograms for ALBA4, T_0_ line Pv_Zm31‐6A, haploid individuals H12.4 and HF11, and three aneuploid individuals. The aneuploid's coverage data indicate approximately 50% of the normalized ALBA4 read counts for specific chromosomes. Aneuploid A149 was monosomic for two chromosomes, while aneuploids A15 and A161 were monosomic for five and eight chromosomes, respectively. Among all 18 sequenced aneuploid individuals, 12 chromosomes were monosomic in at least one line and another three chromosomes contained major deletions in at least one line. Some genome regions in most lines contained more counts than would be expected relative to ALBA4, which appear as peaks in Figure 4b–d. Count data for all 18 lines is included in Table S7.

**FIGURE 5:**
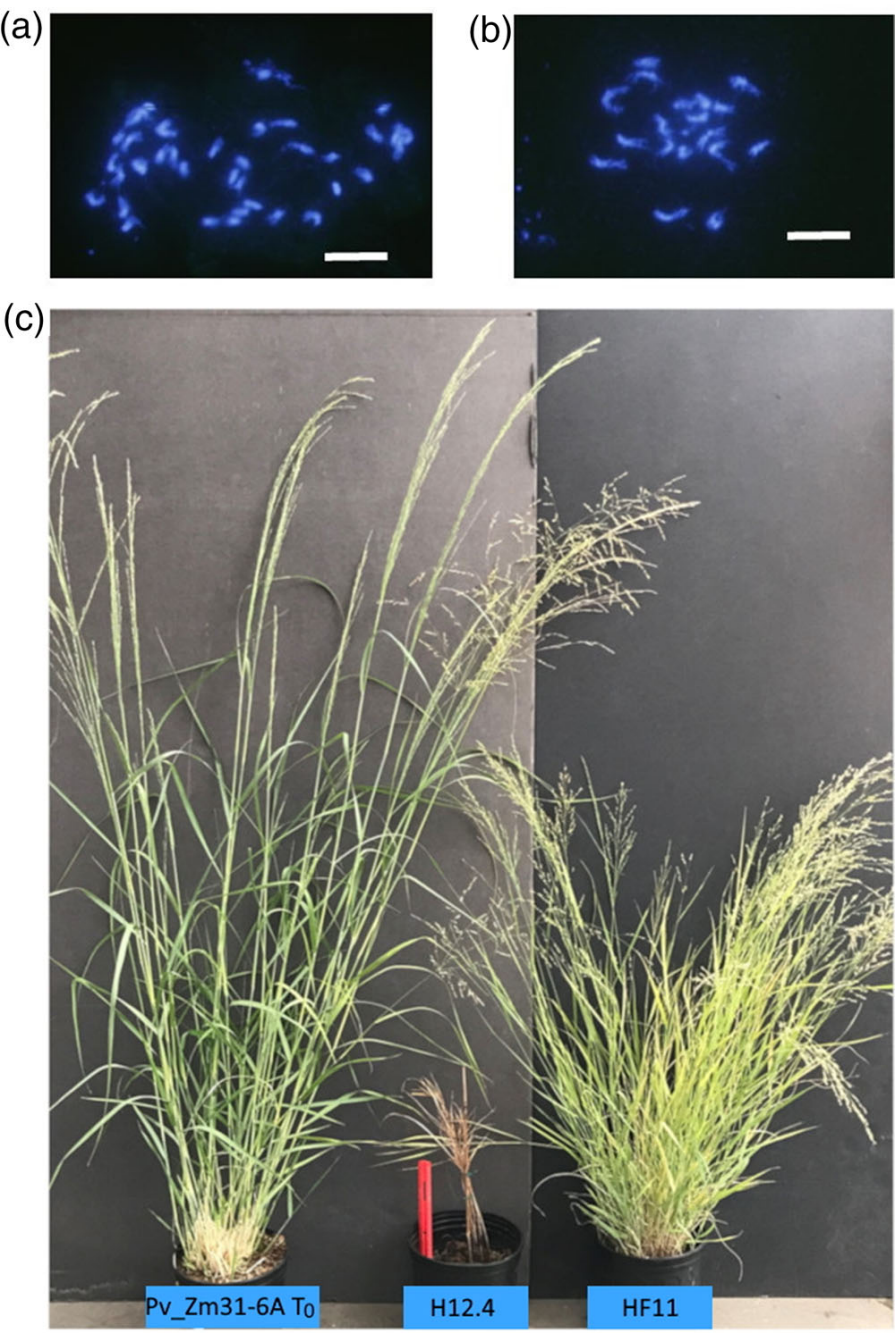
Chromosome squashes (a, b) prepared from root tip cells confirmed 2n  =  36 chromosomes in the tetraploid genotype ALBA4 (a) and 2n  =  18 in dihaploid line HF11(b). Scale bars  =  10 μm. Image in Panel c shows the Pv_Zm31‐6A T_0_ mutant line (left) next to two haploid T1 progeny, H12.4 (middle) and HF11 (right), produced in a cross with AP13. Ruler = 30.5 cm (12 inches)

Metaphase chromosome squashes obtained from root tips and overall growth of the two haploids that were recovered are presented in Figure 5. Two of the haploid progeny grew sufficiently to transfer them to the greenhouse, Haploid H12.4 grew poorly (Figure 5c) and we were unable to obtain healthy root tips from this line. Haploid HF11 grew more vigorously but not as well as the T_0_ line it was derived from, and we verified chromosome counts for this line to be 18.

## 4 DISCUSSION

The generation of DH lines for plant breeding purposes must be efficient and scalable to enable selection on large populations of individuals. In switchgrass, the technology is nonexistent but would be beneficial if it were combined with hybrid breeding schemes that provided superior gains by taking advantage of heterosis. With 18 pairs of individual small chromosomes, switchgrass represents a species that is cytologically between maize (10 pairs) and wheat (21 pairs). Given that haploid‐induction rates can approach 8% in wheat we did not believe this would be a primary determinant of success.

We explored the potential of centromere‐mediated genome elimination with the added step of using CRISPR/Cas9 to effectively induce lesions in two homoeologous *CENH3* genes. We hypothesized that these genes were functionally redundant based on their high degree of amino‐acid identity and expression patterns and would both require targeting with proper sgRNA design. For that reason, we used T‐DNA's directing the expression of a PTG array for producing eight different sgRNA specific for either CENH3‐1 or CENH3‐2.

The resulting transgenic lines from four different CENH3* expression constructs produced at least some aneuploids while the construct where the N‐terminal tail of CENH3‐1 was swapped with that of maize yielded 2 independent lines that induced haploids at low frequency. One of the constructs that produced aneuploids contained a CENH3* which encoded WT CENH3‐2.

In maize, it was determined that an *ImmuneCENH3* complementation gene was not necessary for haploid induction (Wang et al., 2021). Rates of 5% haploid induction were observed when crossing a +/*cenh3* line to a tester line. The mechanism is proposed to involve CENH3 dilution during post‐meiotic nuclear divisions in the gametophyte. Similarly, in wheat haploid inducing lines were produced via genome editing where expression of complementation genes was not required. Although there are three homoeologs of two different *CENH3* genes in wheat, viable +/cenh3 lines could be generated by genome editing alone which contained essentially one WT and one hypomorphic copy of *CENH3*. These also had high rates of haploid induction (8%). If this general approach is possible in switchgrass, it would be consistent with our findings that a specific CENH3* complementation gene was not required for aneuploid induction. Further engineering or designed crosses of existing lines with desired cenh3 alleles are likely to yield new generations with improved haploid induction rates and resolve the possible required activity of a complementation gene. A second reason for poor rates of haploid production is possibly related to inbreeding depression. Unlike wheat or maize, switchgrass maintains high levels of heterozygosity enforced through genetic self‐incompatibility that causes stigmatic inhibition of self‐pollen. Such breeding systems typically maintain higher frequencies of recessive deleterious mutations. In the haploid state many of these could have embryo‐lethal phenotypes. We observed low survival rates in most of the haploids that were detected so it is likely that many others passed detection due to low viability.

Clonal propagation of edited lines from single axillary buds was able to clear up genotyping uncertainty (Table S4). However, the persistent presence of the Cas9 nuclease and PTG array likely creates potential for recurrent target site recognition and cleavage, and thus genotypes of the T_0_ are subject to instability even though nonhomologous end joining DNA repair is a one‐way process. This is a strong rationale for identifying and advancing null T‐DNA segregants containing *CENH3‐1/CENH3‐2* edits. Another rationale is that Cas9/sgRNA ribonucleoprotein complexes may persist in developing T_1_ embryos post‐fertilization and possibly damage the paternal genome. This may reduce recovery of haploids that would otherwise have been viable. Overall, the editing activity that was detected appeared slightly biased toward *CENH3‐2* (Table 2). This result may indicate CENH3‐2 activity is not as essential or is partially compensated by CENH3‐1.

The specific version of CENH3* included in a transformation appeared to influence transformation rates particularly with Pv_GFP31 and Pv_GFP_TS, the two GFP containing constructs. These constructs did not lead to discovery of any lines that produced either haploids or aneuploids. This is interesting because Pv_GFP_TS was designed to be a switchgrass version of the original Arabidopsis *GFP–tailswap* (Ravi & Chan, 2010) and was also found to be transcribed in the one line analyzed.

Sequencing of aneuploids produced expected and some unexpected results. While individuals were monosomic, or partially monosomic for specific chromosomes as expected, there were also genome regions in the sequencing data that were overrepresented relative to ALBA4. These regions could have been amplified through the replication and movement of transposable elements activated during the tissue culture process and then heritably passed to the T_1_. They are present in chromosomes 1K, 7N, and 8K of most aneuploids (Figure 4b–d, data not shown). These duplicated regions may also comprise results of large‐scale rearrangements or translocations that may have occurred. Evidence for these types of rearrangements would require longer sequence reads or detailed cytogenetic analysis.

The transgenic lines we have generated and analyzed here provide an initial attempt and stable resource for exploring the potential of aneuploidy and haploidy in switchgrass breeding. Altogether, eight independent T_0_ lines produced significant numbers of aneuploids and two of these produced haploids. Cases of complete haploidy were rare perhaps due to presence of recessive embryo‐lethal genes, Cas9/sgRNA activity in developing T_1_ embryos, or partial CENH3 activity. Further advances in efficiency and greater understanding of the processes producing haploids may be possible by manipulation of complete or partial loss of function *cenh3* alleles generated here independently of the CENH3* chimeric genes and CRISPR machinery.

## AUTHOR CONTRIBUTIONS

Sangwoong Yoon: Conceptualization; Investigation; Supervision. Jennifer Bragg: Investigation; Writing – original draft; Writing – review & editing. Prisca Cheng: Investigation. Lisa Chanbusarakum: Investigation; Writing – review & editing. Kurtis Dluge: Investigation. Sheyla Aucar‐Yamato: Investigation. Eduardo Blumwald: Funding acquisition; Project administration; Supervision. Yong Gu: Funding acquisition; Project administration; Supervision. Christian M. Tobias: Conceptualization; Resources; Supervision; Writing – review & editing.

## CONFLICT OF INTEREST

The authors declare no conflict of interest.

## Supporting information


**Figure S1**. Conceptual translations of synthetic complementation genes.

**Figure S2**. Locations of primers used in sequencing of CENH3 edits and qRT‐PCR of endogenous CENH3 genes.

**Figure S3**. Expression of complementation transgenes in leaf tissue of transgenic lines.

**TableS 1**: Summary of sequence variants within the *CENH3* coding regions of switchgrass ALBA4 genotype relative to P. virgatum v.5.1 reference sequences.

**Table S2**. Oligonucleotide primers used in this study.

**Table S3**. Summary of *PviCENH3* gene edits in T_0_ lines

Supplementary Material

Supplementary Material

**Table S6. Pv_Zm31** crosses

Supplementary Material
