# Supplementary material for: Haploidy and aneuploidy in switchgrass mediated by misexpression of CENH3
Source: Plant Genome. 2022 Apr 25;16(2):e20209. doi: 10.1002/tpg2.20209 (PMC12807150; doi:10.1002/tpg2.20209)
Supplement: Supplementary file 1 [file TPG2-16-e20209-s002.docx]

| ***Table S2. Oligonucleotide primers used in this study.*** | | |
| --- | --- | --- |
| ***Name*** | **Sequence 5'-3'** | **Purpose** |
| *TG 00 FWD* | TAGGTCTCTGGCAAACAAAGCACCAGTG | PTG construction |
| *TG 00 RVS* | CGGGTCTCACTCACGGCCGCGTGCACCAGCCGGG | PTG construction |
| *TG 01 FWD* | CGGGTCTCATGAGGAAATCCAGTTTTAGAGCTAGAA | PTG construction |
| *TG 01 RVS* | CGGGTCTCAAGCGCCCGAACTTGCACCAGCCGGG | PTG construction |
| *TG 02 FWD* | TAGGTCTCTCGCTCCCCGAACGTTTTAGAGCTAGAA | PTG construction |
| *TG 02 RVS* | CGGGTCTCAGCCCGCTCCCCGTGCACCAGCCGGG | PTG construction |
| *TG 03 FWD* | TAGGTCTCTGGGCTTACCTGTGTTTTAGAGCTAGAA | PTG construction |
| *TG 03 RVS* | CGGGTCTCAACGGTGCGGTTTTGCACCAGCCGGG | PTG construction |
| *TG 04 FWD* | TAGGTCTCTCCGTTGGAGGCCGTTTTAGAGCTAGAA | PTG construction |
| *TG 04 RVS* | CGGGTCTCAGGAAATCGAAGGTGCACCAGCCGGG | PTG construction |
| *TG 05 FWD* | TAGGTCTCTTTCCTCACGGCCGTTTTAGAGCTAGAA | PTG construction |
| *TG 05 RVS* | CGGGTCTCAGCGCCCGAACTGTGCACCAGCCGGG | PTG construction |
| *TG 06 FWD* | TAGGTCTCTGCGCTCCCCGCAGTTTTAGAGCTAGAA | PTG construction |
| *TG 06 RVS* | CGGGTCTCACACTCACCGGAGTGCACCAGCCGGG | PTG construction |
| *TG 07 FWD* | TAGGTCTCTAGTGCGTCCGAGGTTTTAGAGCTAGAA | PTG construction |
| *TG 07 RVS* | TAGGTCTCTAAACACGGTGTGGTTTCTTCACCTTGCACCAGCCGGG | PTG construction |
| *CEN3_2 RVS* | ATGCACTAGTTCACCAGCGCCTTCCA | immunized *CENH3-2* Gblocks cloning |
| *hyg left* | CCCAAGCTGCATCATCGAAA | T0 confirmation |
| *hyg right* | GGAAGTGCTTGACATTGGGG | T0 confirmation |
| *CAS9 400 FWD* | CGTGATCACCGACGAGTACAA | T0 confirmation |
| *CAS9 400 RVS* | GGTGCTGTCCACCAGTTTCTT | T0 confirmation |
| *AscI – AvrII adapter sense strand* | GCATCCTAGGGCATGCATGGCGCGCCGCAT | AscI -AvrII adapter oligo |
| *AscI – AvrII adapter anti-sense strand* | ATGCGGCGCGCCATGCATGCCCTAGGATGC | AscI -AvrII adapter oligo |
| *M Ubi pro FWD* | ATGCCCTGCAGGCTGCAGTGCAGCGTG | Maize ubiquitin promoter cloning |
| *M Ubi pro RVS* | ATGCTTCGAACTGCAGAAGTAACACCAAACAA | Maize ubiquitin promoter cloning |
| *Pvi cassette amp forward* | ATGCGGCGCGCCTCCCTGAGTCCGTTGC | complementation gene cloning |
| *Pvi cassette amp reverse* | GCATCCTAGGTGACAGCTTATCATCGGATCT | complementation gene cloning |
| *Pvi311_1234_F* | CACCGTCCGCCCGAGAGTTC | *CENH3-1* specific primers for genotyping |
| *Pvi311_1234_R* | ACACGACGTACCCAAACCTCACA | *CENH3-1* specific primers for genotyping |
| *GSP2F* | CCCAACCCCTGACAGTTCA | *CENH3-2* specific primers for genotyping |
| *GSP2R* | ATTCAAGCTCACAATGGAGCAAT | *CENH3-2* specific primers for genotyping |
| *Pv31_123 New FWD* | CGAGAGTTCGAACCAAAA | *CENH3-1* specific primers for genotyping |
| *Pv31_4_RVS* | CAATTAGGTACAAGGGATTTAACA | *CENH3-1* specific primers for genotyping |
| *Pv32 new 5678 Fwd* | CCCCTGACAGTTCAAACCCA | *CENH3-2* specific primers for genotyping |
| *GSP1R* | CACATTCCCCCTTGAGTAGAAC | *CENH3-2* specific primers for genotyping |
| *GAPDH CSY 1* | GGAATCAACGGTTTCGGAAGGAT | Pvi qPCR housekeeping gene |
| *GAPDH CSY 2* | ACCGAAGAGAAGGGTTTTGGA | Pvi qPCR housekeeping gene |
| *Cas91* | GGACTCCCGGATGAACACTA | *Cas9* transgene qRTPCR |
| *Cas92* | TCGCTTTCCAGCTTAGGGTA | *Cas9* transgene qRTPCR |
| *31_Endo1F* | GGCCGTGAGGAAATCCAAGGA | Endogenous *CENH3-1* qRTPCR |
| *31_Endo4R* | CTCCAACGGTGCGGTTTC | Endogenous *CENH3-1* qRTPCR |
| *32_EndoF_3* | AGGTGAAGAAACCACACCGTTGGAA | Endogenous *CENH3-2* qRTPCR |
| *32_EndoR_3* | GCTGTATGTCCCTTTGCATGATGGTAAC | Endogenous *CENH3-2* qRTPCR |
| *5_GFPCEN1* | CGCTCAGTAAAGACCCGAAC | eGFP:CENH3-1 qRTPCR |
| *5_GFPCEN2* | CtCCCGAAtTGGAGCTTCTT | eGFP:CENH3-1 qRTPCR |
| 6_ZmCEN1 | GGAGAAGCCCAAGAAGAAGC | Zm:CENH3-1 qRTPCR |
| 6_ZmCEN2 | CAACTCGGTGGATTTCTGGT | Zm:CENH3-1 qRTPCR |
| 7_AtCENH1 | GCGGTGAAGGTGGAGATAAT | At:CENH3-1 qRTPCR |
| *7_AtCENH2* | CAACTCGGTGGATTTCTGGT | At:CENH3-1 qRTPCR |
| *8_Tailswap1* | CCAGCAGAACACACCGATAG | GFP-tailswap qRTPCR |
| *8_Tailswap2* | CCACCTCCTCCACCTTTGTA | GFP-tailswap qRTPCR |
| *32_Imm8_F* | CGGTGGGGCTTCTTAACCTTC | Immunized *CENH3-2* qRTPCR |
| *32_Imm5_R* | CAGCCGTTAGGAAATCAAAGGAGC | Immunized *CENH3-2* qRTPCR |
| *123F_new* | CTGCTGCCTCTTGTATCGCA | Immunized *CENH3-1* qRTPCR |
| *123R_new* | GAGGGTGAAGAAGCCGCATA | Immunized *CENH3-1* qRTPCR |
