# Supplementary material for: Haploidy and aneuploidy in switchgrass mediated by misexpression of CENH3
Source: Plant Genome. 2022 Apr 25;16(2):e20209. doi: 10.1002/tpg2.20209 (PMC12807150; doi:10.1002/tpg2.20209)
Supplement: Supplementary file 2 [file TPG2-16-e20209-s004.docx]

**Table S6. Pv_Zm31** crosses

| **Cross direction** | **Seed/tiller** | **Total seeds** | **100 seed (mg)** | **% germination** |
| --- | --- | --- | --- | --- |
| Cross 1 |  |  |  |  |
| AP13 ♀ x Pv_Zm31-6A ♂ | 462 | 1387 | 93.8 | 70.4 |
| Pv_Zm31-6A ♀ x AP13 ♂ | 125 | 250 | ND | 66.8 |
| Cross 2 |  |  |  |  |
| AP13 ♀ x Pv_Zm31-6A ♂ | 69 | 275 | 98.5 | 91.6 |
| Pv_Zm31-6A ♀ x AP13 ♂ | 83 | 333 | 92.2 | 78 |
