# Supplementary material for: Haploidy and aneuploidy in switchgrass mediated by misexpression of CENH3
Source: Plant Genome. 2022 Apr 25;16(2):e20209. doi: 10.1002/tpg2.20209 (PMC12807150; doi:10.1002/tpg2.20209)
Supplement: Supplementary file 3 [file TPG2-16-e20209-s005.docx]

Immunized CENH3-1 (Pv_control31/Pv_Imm31)

```
MARTKHAAVR KSKEQPKKKL QFGRSPNRRA TPTGGASTSA TPARAAGTGE RAAAGAGTAG  60
RQQQRVKKPH RWRPGTVALR EIRKYQKSTE LLIPFAPFAR LVREITDFYS RGNVTRWTPE 120
ALLAIQEAAE FHLIDLFEVA NLCAIHAKRV TIMQKDMQLA RRIGGRHW              168
```

CENH3-1/G75E (Pv_G75E31)

```
MARTKHAAVR KSKEQPKKKL QFGRSPNRRA TPTGGASTSA TPARAAGTGE RAAAGAGTAG  60
RQQQRVKKPH RWRPETVALR EIRKYQKSTE LLIPFAPFAR LVREITDFYS RGNVTRWTPE 120
ALLAIQEAAE FHLIDLFEVA NLCAIHAKRV TIMQKDMQLA RRIGGRHW              168
```

Immunized CENH3-2 (Pv_Imm32)

```
MARTKHPAVR KSKEQPKKKL QFGRSPHGRA TPTGGASTSA TPASAAGTGE RAAAGAGTAG  60
PQQQKVKKPH RWKPGTVALR EIRKFQKSTE MLIPFAPFAR LVREITEFYS RGNVTRWTPE 120
AILAIQEAAE FHLIELFEVA NLCAIHAKRV TIMQRDIQLA RRIGGRRW              168
```

GFP:CENH3-1 (Pv_GFP31)

```
MVSKGEELFT GVVPILVELD GDVNGHKFSV SGEGEGDATY GKLTLKFICT TGKLPVPWPT  60
LVTTLTYGVQ CFSRYPDHMK QHDFFKSAMP EGYVQERTIF FKDDGNYKTR AEVKFEGDTL 120
VNRIELKGID FKEDGNILGH KLEYNYNSHN VYIMADKQKN GIKVNFKIRH NIEDGSVQLA 180
DHYQQNTPIG DGPVLLPDNH YLSTQSALSK DPNEKRDHMV LLEFVTAAGI TLGMDELYKG 240
GGGGGGTMAR TKHAAVRKSK EQPKKKLQFG RSPNRRATPT GGASTSATPA RAAGTGERAA 300
AGAGTAGRQQ QRVKKPHRWR PGTVALREIR KYQKSTELLI PFAPFARLVR EITDFYSRGN 360
VTRWTPEALL AIQEAAEFHL IDLFEVANLC AIHAKRVTIM QKDMQLARRI GGRHW      415
```

Zm:CENH3-1 (Pv_Zm31)

```
MARTKHQAVR KTAEKPKKKL QFERSGGAST SATPERAAGT GGRAASGGDS VKKTKPRHRW  60
RPGTVALREI RKYQKSTELL IPFAPFARLV REITDFYSRG NVTRWTPEAL LAIQEAAEFH 120
LIDLFEVANL CAIHAKRVTI MQKDMQLARR IGGRHW                           156
```

Ath:CENH3-1 (Pv_Ath31)

```
MARTKHRVTR SQPRNQTDAA GASSSQAAGP TTTPTRRGGE GGDNTQQTNP TTSPATGTRR  60
GAKRSRQAMP RGSQKKSYRY RPGTVALREI RKYQKSTELL IPFAPFARLV REITDFYSRG 120
NVTRWTPEAL LAIQEAAEFH LIDLFEVANL CAIHAKRVTI MQKDMQLARR IGGRHW     176
```

GFP-tailswap (Pv_GFP_TS)

```
MVSKGEELFT GVVPILVELD GDVNGHKFSV SGEGEGDATY GKLTLKFICT TGKLPVPWPT  60
LVTTLTYGVQ CFSRYPDHMK QHDFFKSAMP EGYVQERTIF FKDDGNYKTR AEVKFEGDTL 120
VNRIELKGID FKEDGNILGH KLEYNYNSHN VYIMADKQKN GIKVNFKIRH NIEDGSVQLA 180
DHYQQNTPIG DGPVLLPDNH YLSTQSALSK DPNEKRDHMV LLEFVTAAGI TLGMDELYKG 240
GGGGGGTMAR TKQTARKSTG GKAPRKQLAT KAARKSAPAT GGVKKPHRFR PGTVALREIR 300
KYQKSTELLI PFAPFARLVR EITDFYSRGN VTRWTPEALL AIQEAAEFHL IDLFEVANLC 360
AIHAKRVTIM QKDMQLARRI GGRHW                                       385
```

**Figure S1.** Conceptual translations of synthetic complementation genes. Each complementation peptide and the vectors (in parentheses) where they are present is listed. All synthetic complementation genes were placed between 2 kb of CENH3-2 upstream regulatory region and the Nos terminator to drive their expression. Highlighted residues are single amino-acid alterations relative to CENH3-1 peptide. Colors indicate: G75E mutation, light blue; AA substitutions in CENH3-2 relative to CENH3-1, gray; eGFP, green; glycine linker, red; N-terminal tail of *Z. maize* CENH3, light brown; N-terminal tail of *A. thaliana* CENH3, blue; N-terminus of *P. virgatum* conventional H3, purple.
