# Supplementary material for: Haploidy and aneuploidy in switchgrass mediated by misexpression of CENH3
Source: Plant Genome. 2022 Apr 25;16(2):e20209. doi: 10.1002/tpg2.20209 (PMC12807150; doi:10.1002/tpg2.20209)
Supplement: Supplementary file 4 [file TPG2-16-e20209-s007.docx]

| **TableS 1:** Summary of sequence variants within the *CENH3* coding regions of switchgrass ALBA4 genotype relative to P. virgatum v.5.1 reference sequences. Results were tabulated after alignment of Illumina Hiseq reads from JGI project ID: 1017141, *P. virgatum* A4 Resequencing. | | | | | | | | |
| --- | --- | --- | --- | --- | --- | --- | --- | --- |
| ***CenH3-1*** | | | | | | | | |
| **Change^†^** | **Polymorphism Type** | **Variant Freq. ^‡^** | **P-value** | **AA Change** | **CDS Position** | **Codon Change** | **Protein Effect** | **Phase Blocks^§^** |
| C -> T | transition | 60.00% | 3.30E-12 | None | 435 | ATC -> ATT | silent | 0\|1 |
| A -> G | transition | 33.80% | 3.70E-13 | K -> R | 464 | AAG -> AGG | conservative | 0\|2 |
| G -> A | transition | 33.30% | 4.10E-51 | M -> I | 471 | ATG -> ATA | conservative | 0\|2 |
| AT -> GC | Substitution | 25.90% | 7.00E-22 | H -> R | 500 | CAT -> CGC | conservative | 0\|2 |
| ***CenH3-2*** | | | | | | | | |
| C -> A | transversion | 27.30% | 9.50E-07 | H -> N | 79 | CAC -> AAC | conservative | 0\|1 |
| G -> A | transition | 47.60% | 3.50E-24 | None | 177 | GCG -> GCA | silent | 0\|2 |
| C -> G | transversion | 47.60% | 3.30E-16 | P -> R | 182 | CCG -> CGG | neutral small > hydrophilic large | 0\|2 |
| A -> G | transition | 46.20% | 9.10E-11 | K -> R | 194 | AAG -> AGG | conservative | 0\|2 |
| A -> G | transition | 48.50% | 1.10E-39 | None | 207 | CCA -> CCG | silent | 0\|2 |
| A -> G | transition | 53.80% | 5.20E-32 | K -> R | 218 | AAG -> AGG | conservative | 0\|2 |
| T -> A | transversion | 50.00% | 1.20E-56 | F -> Y | 254 | TTC -> TAC | conservative | 0\|2 |
| A -> T | transversion | 48.20% | 2.50E-44 | M -> L | 271 | ATG -> TTG | conservative | 0\|2 |
| G -> C | transversion | 47.80% | 3.30E-87 | E -> D | 321 | GAG -> GAC | conservative | 0\|2 |
| G -> A | transition | 45.50% | 4.40E-48 | None | 342 | GTG -> GTA | silent | 0\|2 |
| CA -> TC | Substitution | 42.9% -> 44.9% | 2.50E-80 | AI -> AL | 363 | GCC,ATC -> GCT,CTC | conservative | 0\|2 |
| A -> G | transition | 44.90% | 2.00E-83 | None | 372 | GCA -> GCG | silent | 0\|2 |
| A -> T | transversion | 30.40% | 9.50E-40 | E -> D | 405 | GAA -> GAT | conservative | 0\|3 |
| C -> T | transition | 28.00% | 1.50E-33 | None | 453 | ACC -> ACT | silent | 0\|3 |
| C -> A | transversion | 26.50% | 1.00E-16 | None | 456 | ATC -> ATA | silent | 0\|3 |
| **^†^**All sites were heterozygous for reference allele and alternative allele.  **^‡^** Sites were covered by a minimum of 10 reads.  **^§^** Variant sites linked to the same haplotype by phase-informative short reads are shown with the same code. 0 = ref. allele. | | | | | | | | |
