# Supplementary material for: Haploidy and aneuploidy in switchgrass mediated by misexpression of CENH3
Source: Plant Genome. 2022 Apr 25;16(2):e20209. doi: 10.1002/tpg2.20209 (PMC12807150; doi:10.1002/tpg2.20209)
Supplement: Supplementary file 5 [file TPG2-16-e20209-s010.docx]

**Table S3.** Summary of *PviCENH3* gene edits in T_0_ lines

|  |  |  |  | **T_0_ CENH3-1** | | | **T_0_ CENH3-2** | | |
| --- | --- | --- | --- | --- | --- | --- | --- | --- | --- |
| **Construct** | **# lines produced** | **# lines genotyped** | **# lines with mutations** | **no edits (%)†** | **HM edits (%)** | **HT edits (%)** | **no edits (%)** | **HM edits (%)** | **HT edits (%)** |
| Pv_control31 | 3 | 3 | 0 | 3 (100) | 0 (0) | 0 (0) | 3 (100) | 0 (0) | 0 (0) |
| Pv_Imm31 | 13 | 10 | 1 | 9 (90) | 0 (0) | 1 (10) | 10 (100) | 0 (0) | 0 (0) |
| Pv_G75E31 | 5 | 1 | 1 | 1 (100) | 0 (0) | 0 (0) | 0 (0) | 0 (0) | 1 (100; chimeric plant, edit not fixed) |
| Pv_Imm32 | 17 | 8 | 5 | 7 (88) | 1 (12) | 0 (0) | 3 (33) | 4 (56) | 1 (11) |
| Pv_GFP31 | 1 | 1 | 1 | 1 (100) | 0 (0) | 0 (0) | 0 (0) | 0 (0) | 1 (100) |
| Pv_Zm31 | 13 | 10 | 9 | 5 (50) | 1 (10) | 4 (40) | 2 (20) | 5 (50) | 3 (30; 1 non-frameshift) |
| Pv_Ath31 | 7 | 6 | 5 | 5 (83) | 1 (17) | 0 (0) | 1 (17) | 2 (33) | 3 (50; all non-frameshift) |
| Pv_GFP_TS | 2 | 2 | 2 | 0 (0) | 1 (50) | 1 (50) | 0 (0) | 2 (100) | 0 (0) |

†Percent of genotyped lines showing the listed edit types for each gene

HM = homozygous, HT = heterozygous
